# Meta-Analysis of Therapy of Cinobufacini Capsule Adjunct with First-Line Platinum-Based Chemotherapy for the Treatment of Advanced NSCLC

**DOI:** 10.1155/2021/5596415

**Published:** 2021-08-21

**Authors:** Wenpan Peng, Yong Xu, Fanchao Feng, Cheng Gu, Zhichao Wang, Di Han, Xianmei Zhou, Hailang He

**Affiliations:** ^1^Affiliated Hospital of Nanjing University of Chinese Medicine, Nanjing 210029, China; ^2^Department of Respiratory Medicine, Jiangsu Province Hospital of Chinese Medicine, Nanjing 210029, China

## Abstract

**Background:**

Cinobufacini capsule, an anticancer traditional Chinese patent medicine, has been widely used as adjunctive treatment to platinum-based chemotherapy in patients with advanced NSCLC.

**Purpose:**

To evaluate the efficacy and safety of cinobufacini capsule combined with first-line platinum-based chemotherapy for advanced NSCLC. *Study Design*. A systematic review and meta-analysis of eight outcome measures selected for this study were performed according to the Preferred Reporting Items for Systematic Reviews and Meta-Analyses (PRISMA) guidelines.

**Methods:**

A comprehensive literature search was conducted in 7 electronic databases to identify all the relevant randomised controlled trials. Cochrane handbook 5.1.0 was applied to evaluate the quality of included trials, and the RevMan 5.3 and Stata 15.1 software were used to combine the trials for data analysis and assess the publication bias.

**Results:**

From the 19 studies reviewed, a total of 1,564 patients were included. Compared with first-line platinum-based chemotherapy alone, cinobufacini capsule combined with chemotherapy showed significant effects in improving ORR (RR = 1.49, 95% CI (1.33, 1.66)), 1-year survival rate (RR = 1.44, 95% CI (1.28, 1.63)), and 2-year survival rate (RR = 1.78, 95% CI (1.42, 2.22)), raising the percentages of CD3^+^ cells (SMD = 1.25, 95% CI (1.05, 1.45)), CD4^+^ cells (SMD = 1.52, 95% CI (1.33, 1.71)), and ratio of CD4^+^/CD8^+^ (SMD = 1.36, 95% CI (1.17, 1.54)), and reducing chemotherapy toxicity including leukopenia (RR = 0.61, 95% CI (0.51, 0.72)), thrombocytopenia (RR = 0.52, 95% CI (0.41, 0.67)), and vomiting (RR = 0.79, 95% CI (0.70, 0.88)).

**Conclusion:**

Cinobufacini capsule may increase the therapeutic effectiveness, improve cellular immune function, and reduce the toxicity of first-line platinum-based chemotherapy in patients with NSCLC. These results require confirmation by further rigorously designed randomised controlled trials (RCTs).

## 1. Introduction

Lung cancer is the most frequently diagnosed cancer and the leading cause of cancer-related deaths worldwide [1]. For the purposes of treatment, lung cancer is classified as SCLC and NSCLC which accounts for approximately 83% of all lung cancer cases [2]. Because early-stage NSCLC is typically asymptomatic, approximately 61% of patients with NSCLC are diagnosed at an advanced stage and lose the opportunity for surgery [3]. As a potentially curable treatment, platinum-based chemotherapy is still occupying the dominant position in the treatment for advanced NSCLC because of its effectiveness in decreasing the size of tumor [4]. However, although technology continues to advance, chemotherapy for NSCLC is still associated with low efficacy and accompanied with some adverse effects [5]. Some patients even cannot continue the therapy due to serious side effect of chemotherapy. Therefore, seeking a drug that can improve the efficacy and alleviate the toxicity of chemotherapy is extremely essential.

In complementary and alternative medicines, traditional Chinese medicine is one of the popular adjunctive treatments for lung cancer, mainly by enhancing immunity and reducing the adverse effects of chemotherapy [6, 7]. Cinobufacini (also called Huachansu in Chinese) capsule, a formulation of traditional Chinese medicine preparation, is produced from dried toad venom from the skin glands of Bufo and has been approved by the Chinese State Food and Drug Administration (SFDA) for the treatment of a variety of cancers [8]. Our previous systematic review has showed that cinobufacini injection as an adjuvant therapy to platinum-contained chemotherapy can increase survival rate, improve tumor response, and reduce the toxicity of chemotherapy in advanced NSCLC patients [9–11]. Studies have identified that cinobufacini, containing bufalin, resibufogenin, 5-hydroxytryptamine, etc, can inhibit proliferation, promote apoptosis, and increase immunity in the treatment of tumors [12–14].

**Figure 1:**
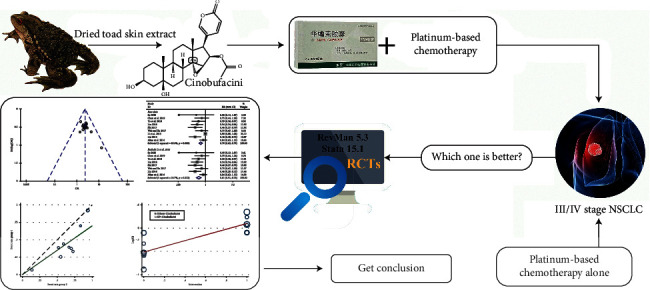
Work flow of the present study.

In recent years, the number of RCTs on cinobufacini capsule combined with chemotherapy for the treatment of NSCLC has increased. A previous meta-analysis indicated that cinobufacini capsule combined with platinum-based chemotherapy might increase efficacy and alleviate the toxicity of chemotherapy for patients with NSCLC [15]. However, only seven RCTs were included in that study, and the methodological quality of the included trials was inadequate. Recently, there have emerged several new clinical trials evaluating the efficacy of cinobufacini capsule combined with platinum-based chemotherapy for NSCLC. Therefore, with an expectation to provide stronger evidence for the clinical application of cinobufacini capsule for NSCLC, this updated systematic review and meta-analysis was conducted to evaluate the efficacy and safety of cinobufacini capsule using Cochrane systemic evaluation methods, by gathering all the related studies (Figure 1).

## 2. Data and Methods

### 2.1. Literature Search Strategy

Four Chinese databases, including the Chinese National Knowledge Infrastructure (CNKI), Wanfang Database, Chinese Scientific Journal Database (VIP), and Chinese Biomedical Literature Database (CBM), as well as three English databases, including PubMed, Cochrane Library, and Embase, were searched for RCT literatures on the treatment of NSCLC using combined first-line platinum-based chemotherapy with cinobufacini capsule. All of those searches ended on Jul 01, 2020, and retrieval terms were “cinobufotalin capsule,” “huachansu capsule,” “non small cell lung cancer,” “non small lung cancer,” “non-small cell cancer,” “non-small cell lung,” “non-small cell lung cancer,” “non-small cell lung cancers,” “non-small-cell lung cancer,” “RCT,” and “randomised control.” The references of pertinent publications were manually searched for additional studies.

### 2.2. Inclusion Criteria

Eligible studies for inclusion in the meta-analysis met the following criteria: (1) patients had to be diagnosed with NSCLC of stage III/IV by histopathological or cytological diagnostic criteria; (2) studies concerning clinical RCTs and the treatment group with cinobufotalin capsule in combination with first-line platinum-based chemotherapy and the control group with chemotherapy alone. (3) The outcome should include at least one of the following indicators: ORR, one-year survival rate, two-year survival rate, leukocyte toxicity, platelet toxicity, vomiting toxicity, CD3^+^ level, CD4^+^ level, and CD4^+^/CD8^+^ level.

### 2.3. Exclusion Criteria

The exclusion criteria were as follows: (1) dissertations, reviews, conference papers, or animal experiments; (2) in the experimental group, cinobufacini was the only drug of treatment; no chemotherapy or other drugs were used in addition to cinobufacini, and no first-line platinum-based chemotherapy drugs were used in the control group; (3) the research method was a nonclinical randomised controlled trial; (4) pathological diagnosis is not stage III/IV or could not be determined; and (5) outcome indicator report documents were not standardised or lacked detail.

### 2.4. Data Extraction and Quality Assessment

The detailed method followed the previously reported one [16]. Two investigators (Wenpan Peng and Yong Xu) independently reviewed the eligible studies and extracted the data. This course was cross checked in order to ensure reliability and accuracy. The following information was collected: authors, title of study, year of publication, study size, age and sex of the participants, details of methodological information, interventions, outcomes, and adverse effects for each study. Any disagreements were resolved by consultation of two other reviewers (Hailang He and Xianmei Zhou).

The methodological quality of the included studies was evaluated independently by two reviewers (Wenpan Peng and Yong Xu). According to the Cochrane handbook version 5.1.0 bias risk scoring system, random sequence generation, allocation concealment, blinding (or masking), incomplete data assessment, selective outcome reporting, and other sources of bias were assessed with three potential responses: yes, no, and unclear [17]. Any disagreements were resolved by discussion with the two other reviewers (Hailang He and Xianmei Zhou).

### 2.5. Outcome Measures

Primary outcome indices of efficacy were short- and long-term efficacy. The long-term efficacy was assessed by 1- and 2-year survival rates, and the short-term efficacy was assessed by using the judgement criteria for solid tumours from the World Health Organisation (WHO) [18], i.e., the ORR (objective response rate) = complete remission (CR) + partial remission (PR). The CD4^+^/CD8^+^ ratio and CD3^+^ and CD4^+^ level in T cells of peripheral blood lymphocytes were assessed as immune and biochemical indicators. Adverse reactions were classified according to the toxicity classification standards of the WHO and divided into grades 0–IV. Grade II–IV toxicities were considered adverse reactions [19]. Secondary outcome indices of efficacy were leukocyte and platelet toxicities, as well as vomiting in the digestive tract.

### 2.6. Statistical Analysis

The meta-analysis was performed using RevMan 5.3 (Copenhagen: the Nordic Cochrane Centre, the Cochrane Collaboration, 2014) and Stata 15.1 (Stata Corp., College Station, TX, USA). During data entry, cross checking was carried out to ensure accurate data entry. We calculated the relative risk (RR) with 95% confidence and SMD to compare dichotomous and continuous variables, respectively. Heterogeneity was assessed using Cochran's *Q* test and Higgins' *I*^2^ indicator, and the test level was *P* > 0.05 and *I*^2^ = 50% [17, 20]. If the heterogeneity existed in pooled studies (*I*^*2*^ > 50%), the random model was applied. Otherwise, the fixed model was used. The potential publication bias was evaluated through funnel plots and assessed by Egger's test [21]. If *P* > 0.05, no publication bias was present. The sensitivity was assessed through deleting the studies with high weight and significant differences.

## 3. Results

### 3.1. Retrieval Results

**Figure 2:**
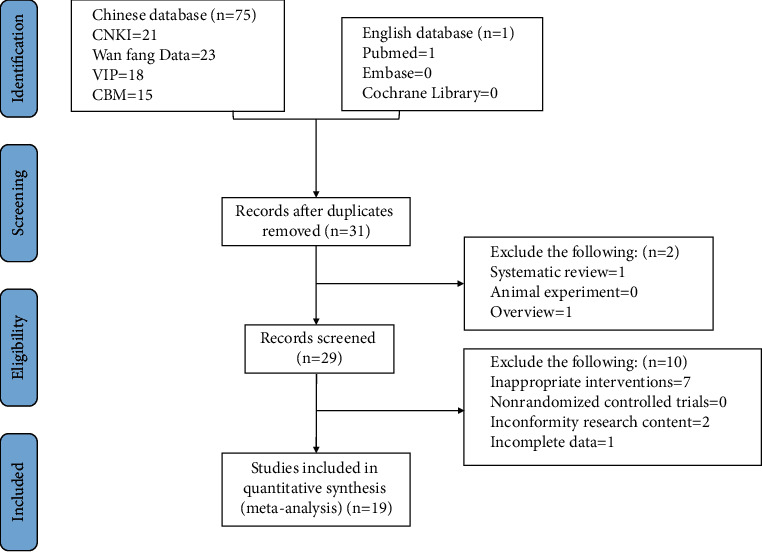
Preferred reporting items for systematic reviews and meta-analysis (PRISMA) search diagram.

The initial search in the electronic database identified 76 potentially relevant studies. A total of 31 records were identified after removing duplicates and screening the titles and abstracts. Twelve trials were excluded for the following reasons: systematic reviews (*n* = 1), animal experiments (*n* = 0), overview (*n* = 1), inappropriate interventions (*n* = 7), non-RCTs (*n* = 0), inconformity research content (*n* = 2), and incomplete data (*n* = 1). Nineteen clinical trials were included in the final meta-analysis. A flowchart describing the literature search and study selection is shown in Figure 2.

### 3.2. Study Characteristics

The characteristics of the 19 RCTs comprising 1,564 patients, with 796 and 768 patients in the experimental and control groups, respectively, are summarized in Table 1 (19 RCTs). All included RCTs were conducted in China, and the articles were published from 2011 to 2020. All patients were divided into two groups, and the clinical diagnosis of all patients was stage III/IV. The intervention for the control group was first-line platinum-based chemotherapy alone, whereas the intervention for the experimental group was cinobufacini capsule combined with the first-line platinum-based chemotherapy regimen.

### 3.3. Study Quality

**Figure 3:**
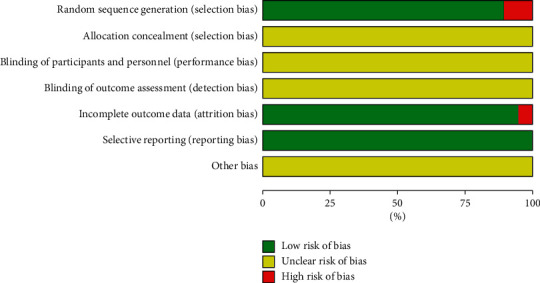
Risk of bias graph.

**Figure 4:**
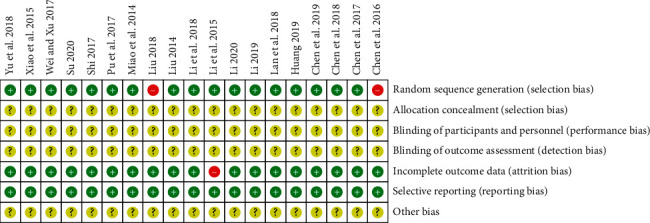
Risk of bias summary.

In all 19 RCTs, 17 of them referred to random number tables or random sequence methods, which were rated as “low risk” and two RCTs were grouped by treatment and rated as “high risk.” None of the 19 articles mentioned allocation concealment and blindness, and the assessment was “unclear.” One of the missing cases, with incomplete report results, was rated as “high risk.” None of the 19 articles had selective reporting bias and were rated as “low risk.” Nineteen articles could not be judged for other-source biases and were rated as “unclear,” as shown in Figures 3 and 4.

### 3.4. Evidence Quality Evaluation

The outcome indicators in this study were graded by using the GRADE evaluation tool. Since all the studies included in this analysis were randomised controlled studies, which were preset to the highest level in the evaluation tool, the quality of the evidence should be considered according to the five downgrade factors. Due to the risk of bias in random concealment, blind, insufficient sample size, and other factors, the outcome indicators were considered to be downgraded, as shown in Table 2.

### 3.5. Meta-Analysis

#### 3.5.1. ORR

**Figure 5:**
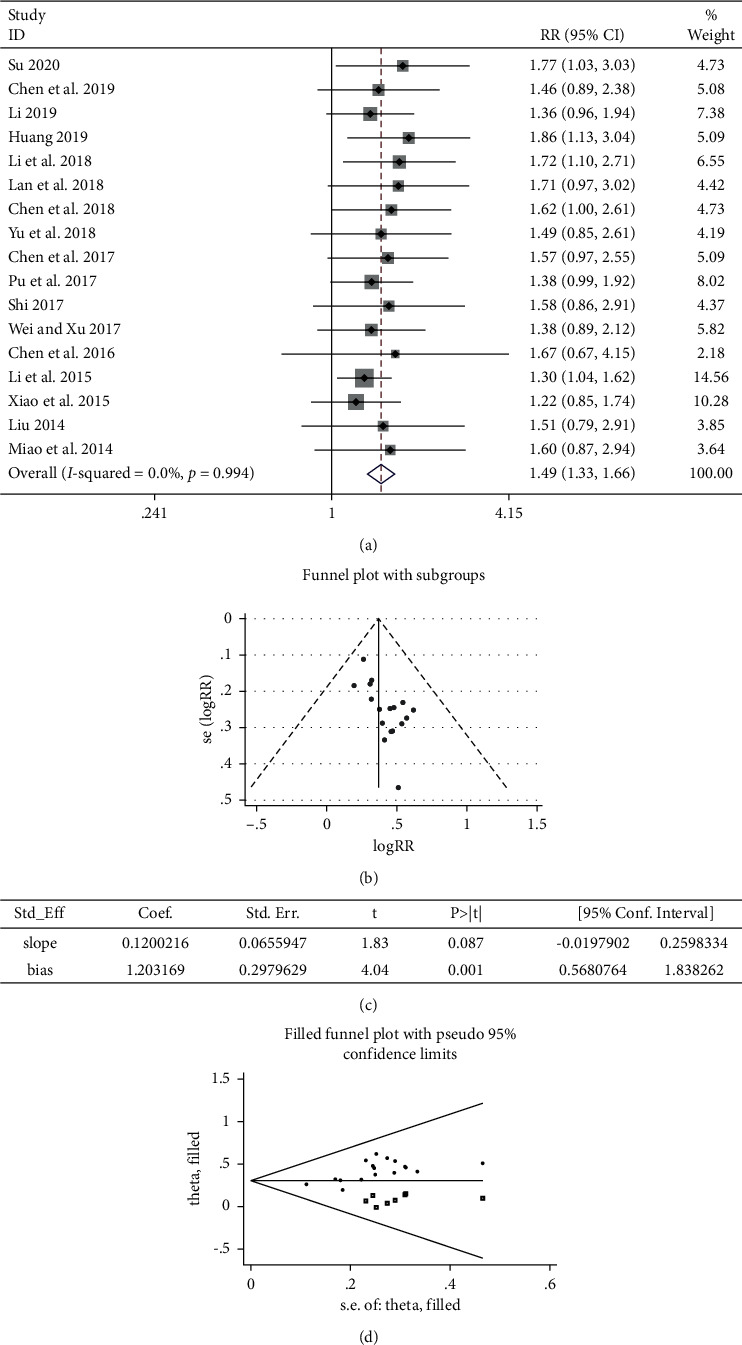
Meta-analysis showing a significant improvement in the ORR in the experimental group compared with that of the control group. (a) Meta-analysis of ORR in included studies. (b) Funnel plots for publication biases of ORR in included studies. (c) Results of Egger's bias test. (d) The impact of publication bias on results by the trim and fill method.

The summary of the meta-analysis is listed in Table 3. The ORR was reported in 17 research articles [22–29, 31–39] with a total of 1,445 patients, including 735 and 710 cases in the cinobufacini capsule plus chemotherapy and chemotherapy alone groups, respectively. The heterogeneity test result was *P* > 0.05 and *I*^2^ = 0.0%, suggesting that no heterogeneity was observed among the results (Figure 5(a)); therefore, the fixed-effect model was applied to combine the trials (RR = 1.49, 95% CI (1.33, 1.66)).

The funnel plot was used to investigate the presence of publication bias in this study (Figure 5(b)), and Egger's bias test indicated that *P* < 0.005 (Figure 5(c)), suggesting that there was a certain publication bias. The funnel plot was processed by the trim and fill method (Figure 5(d)). Therefore, it was necessary to continue to include seven documents whose results were similar to the RCTs [22, 23, 26–28, 33, 39]. This guaranteed the symmetry of the funnel plot and eliminated the publication bias. In summary, compared with chemotherapy alone, the treatment of first-line platinum-based chemotherapy plus cinobufacini capsule could significantly improve ORR.

#### 3.5.2. Survival Rate

**Figure 6:**
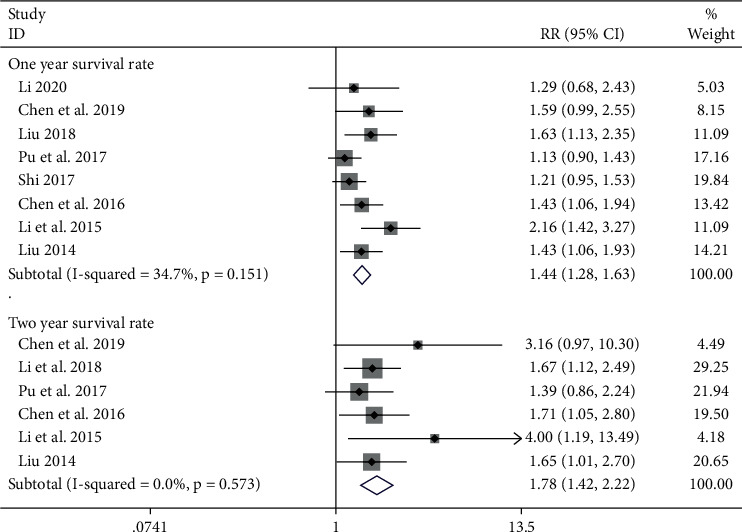
Meta-analysis showing a significant improvement in the survival rate in the experimental group compared with that of the control group.

The 1-year survival rate included 8 studies [24, 30, 32, 33, 35, 36, 38, 40] with a total of 659 patients, including 338 cases in the experimental group and 321 cases in the control group. Six studies [23, 24, 32, 35, 36, 38] including 554 patients that reported the 2-year survival rate were involved. The fixed-effect model combined the trials, 1-year survival rate (RR = 1.44, 95% CI (1.28, 1.63)), and 2-year survival rate (RR = 1.78, 95% CI (1.42, 2.22)). Results of the heterogeneity test results indicated that the 1-year survival rate was *P* > 0.05, *I*^2^ = 34.7% and the 2-year survival rate was *P* > 0.05, *I*^2^ = 0.0% (Figure 6), suggesting that the heterogeneity was acceptable.

#### 3.5.3. Leukocyte Toxicity

**Figure 7:**
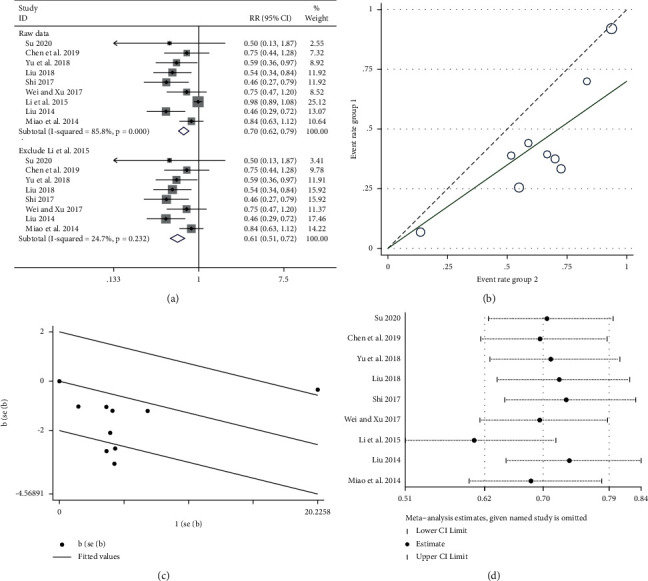
Meta-analysis showing a significant reduction in the leukocyte toxicity in the experimental group compared with that of the control group. (a) Meta-analysis of leukocyte toxicity in included studies. (b, c) The impact of the literature on heterogeneity by the L'Abbe plot and Galbraith plot. (d) The sensitivity analysis of the nine included articles.

In this study, leukocyte decline was reported in 9 RCTs [22, 24, 29, 30, 33, 34, 36, 38, 39], involving a total of 739 patients, including 376 cases in the experimental group and 363 cases in the control group. The heterogeneity test (*P* < 0.005, *I*^2^ = 85.8% (Figure 7(a) upper part)) suggested that there was significant heterogeneity among the RCTs included in this study. Further investigation of the L'Abbe plot (Figure 7(b)) and Galbraith plot (Figure 7(c)) suggested that some articles had a greater impact on heterogeneity. A sensitivity analysis of the 9 included articles revealed that the result of the study by Li et al. [36] had a greater impact on heterogeneity (Figure 7(d)). Meta-analysis was performed after removal of the study using fixed-effect model combined trials (RR = 0.61, 95% CI (0.51, 0.72)). The heterogeneity test (*P* > 0.05, *I*^2^ = 24.7%) (Figure 7(a) lower part) suggested that heterogeneity was within the acceptable range.

#### 3.5.4. Platelet Toxicity

**Figure 8:**
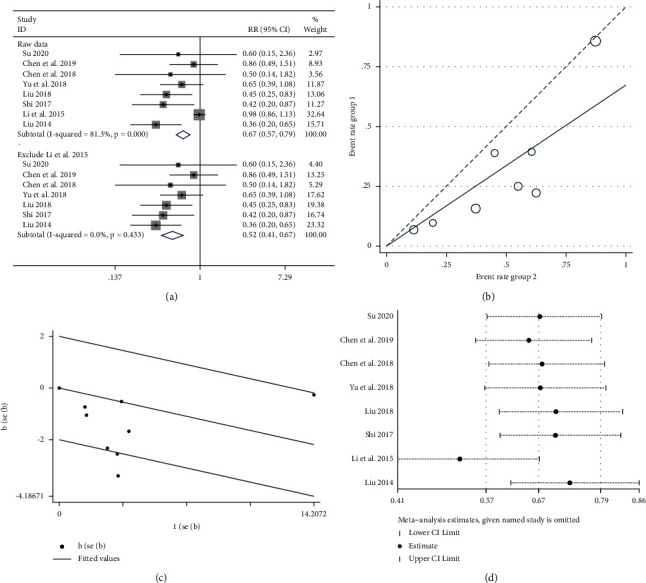
Meta-analysis showing a significant reduction in the platelet toxicity in the experimental group compared with that of the control group. (a) Meta-analysis of platelet toxicity in included studies. (b, c) The impact of the literature on heterogeneity by the L'Abbe plot and Galbraith plot. (d) The sensitivity analysis of the eight included articles.

A total of 8 RCTs [22, 24, 28–30, 33, 36, 38] were included: 676 patients, including 343 cases in the experimental group and 333 cases in the control group. The heterogeneity test result was *P* < 0.005, *I*^2^ = 81.3% (Figure 8(a) upper part), suggesting that there was significant heterogeneity among the RCTs included in this study. Further investigation of the L'Abbe plot (Figure 8(b)) and Galbraith plot (Figure 8(c)) suggested that some articles had a greater impact on heterogeneity. A sensitivity analysis of the 8 included RCTs revealed [36] a greater impact on heterogeneity (Figure 8(d)). Meta-analysis was performed after removal of the study using fixed-effect model combined trials (RR = 0.52, 95% CI (0.41, 0.67)); the heterogeneity test revealed *P* > 0.05, *I*^2^ = 0.0% (Figure 8(a) lower part), and heterogeneity was significantly reduced.

#### 3.5.5. Vomiting Toxicity

**Figure 9:**
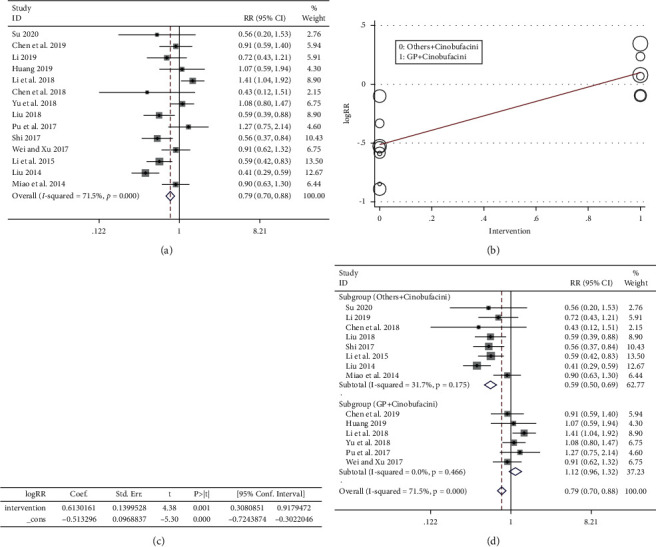
(a) Meta-analysis of vomiting response in included studies. (b, c) The results of the meta-regression analysis. (d) The subgroup study of vomiting toxicity.

The outcome included 14 articles [22–26, 28–30, 32–34, 36, 38, 39], a total of 1162 patients, including 588 cases in the experimental group and 574 cases in the control group. The heterogeneity was *P* < 0.005 and *I*^2^ = 71.5% (Figure 9(a)), suggesting that there was strong heterogeneity among the included literatures in this study. According to Figure 9(a) and Table 1, it was highly suspected that heterogeneity resulted from different intervention measures. The results of the meta-regression analysis of 14 literatures with the ‘logRR' as the dependent variable and ‘intervention' as the independent variable are shown in Figures 9(b) and 9(c), suggesting the independent variable ‘intervention' could significantly affect the trials. Based on this conclusion, a subgroup study was conducted (Figure 9(d)). The result of the heterogeneity test for the subgroup (others + cinobufacini) was *P* > 0.05, *I*^2^ = 31.7%, fixed-effect model combined trials (RR = 0.59, 95% CI (0.50, 0.69)), and the weight was 62.77%. The result of heterogeneity test for the subgroup (GP + cinobufacini) showed that *P* > 0.05, *I*^2^ = 0.0%, the fixed-effect model was used to combine trials (RR = 1.12, 95% CI (0.96, 1.32)), and the weight was 37.23%. In summary, vomiting toxicity was aggravated with GP chemotherapy, while it reduced with other chemotherapy regimens.

#### 3.5.6. Immune Response

**Figure 10:**
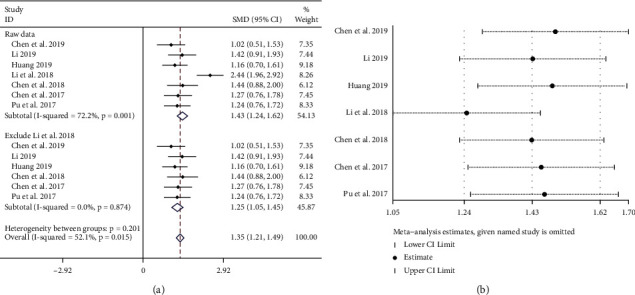
Meta-analysis showing a significant improvement in the CD3^+^ level in the experimental group compared with that of the control group. (a) Meta-analysis of CD3^+^ level in included studies. (b) The sensitivity analysis of the seven included articles.

*(1)*. *CD3*^+^*T Cells*. A total of 558 patients were included in this study, including 284 cases in the experimental group and 274 cases in the control group. The heterogeneity among the 7 articles [23–26, 28, 31,32] included in this study was examined by a forest plot (*P* < 0.005, *I*^2^ = 72.2%; Figure 10(a) upper part), and obvious heterogeneity was noted. Sensitivity analysis was performed on seven articles (Figure 10(b)), and it was highly suspected that heterogeneity was generated by the article by Li et al. [23]. After removing this article, heterogeneity was *P* > 0.05 and *I*^2^ = 0.0% (Figure 10(a) lower part) and significantly reduced. The fixed-effect model combined trials (SMD = 1.25, 95% CI (1.05, 1.45)). In summary, the experimental group can significantly improve the level of CD3^+^.

**Figure 11:**
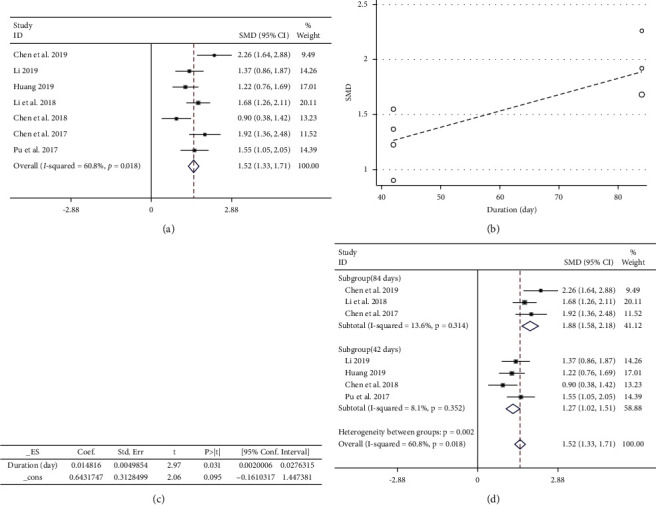
(a) Meta-analysis of CD4^+^ level in included studies. (b, c) The results of the meta-regression analysis. (d) The subgroup study of CD4^+^ level.

*(2)*. *CD4*^+^*T Cells*. A total of 558 patients were included in the outcome, including 284 cases in the experimental group and 274 cases in the control group. The heterogeneity among the 7 RCTs [23–26, 28, 31, 32] included in this study was *P* < 0.05, *I*^2^ = 60.8% (Figure 11(a)), and there was obvious heterogeneity. It was highly suspected that heterogeneity was caused by the difference in the time of administration in the meta-regression analysis of 14 literatures with the ‘SMD' as the dependent variable and ‘duration' as the independent variable; the results are shown in Figures 11(b) and (c). For *P* < 0.05, the independent variable ‘duration' could significantly affect the trials. The result of the heterogeneity test for the subgroup (84 days) was *P* > 0.05, *I*^2^ = 13.6%, the fixed-effect model combined trials (SMD = 1.88, 95% CI (1.58, 2.18)), and the weight was 41.12%. The result of the heterogeneity test for the subgroup (42 days) indicated *P* > 0.05, *I*^2^ = 8.1%; the fixed-effect model was used to combine trials (SMD = 1.27, 95% CI (1.02, 1.51)), and the weight was 58.88% (Figure 11(d)). In summary, the experimental group could significantly improve the CD4^+^ level, and 84 days was more effective than 42 days.

**Figure 12:**
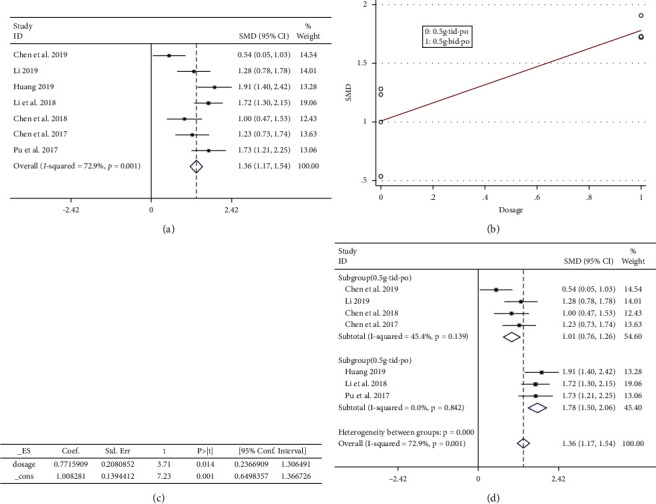
(a) Meta-analysis of CD4^+^/CD8^+^ level in included studies. (b, c) The results of the meta-regression analysis. (d) The subgroup study of CD4^+^/CD8^+^ level.

*(3)*. *CD4*^+^*/CD8*^+^*T-Cell Ratio*. A total of 558 patients were included in the outcome, including 284 cases in the experimental group and 274 cases in the control group. The heterogeneity among the 7 RCTs [23–26, 28, 31, 32] included in this study was *P* < 0.005, *I*^2^ = 72.9% (Figure 12(a)), and there was obvious heterogeneity. We highly suspected that heterogeneity was caused by the difference in the administration dose; then, the ‘SMD' as the dependent variable dose was an independent variable for meta-regression analysis of 7 RCTs. The results are shown in Figures 12(b) and 12(c). For *P* < 0.05, the independent variable ‘administered dose' could significantly affect the “SMD.” A subgroup study was performed (Figure 12(d)). The result of the heterogeneity test for the subgroup (0.5 g·tid·po) was *P* > 0.05, *I*^2^ = 45.4%, the fixed-effect model combined trials (SMD = 1.01, 95% CI (0.76, 1.26)), and the weight was 54.6%; The result of the heterogeneity test for the subgroup (0.5 g·bid·po) indicated *P* > 0.05, *I*^2^ = 0.0, the fixed-effect model was used to combine trials (SMD = 1.78, 95% CI (1.50, 2.06)), and the weight was 45.4%. In summary, the experimental group could significantly increase the CD4^+^/CD8^+^ ratio, and 0.5 g·bid·po was more obvious.

## 4. Discussion

A total of 19 clinical RCTs with 1,564 individuals suffering from advanced NSCLC were selected in this meta-analysis. The main results demonstrated that combining first-line platinum-based chemotherapy with cinobufacini capsule in the treatment of NSCLC may increase survival rate, ORR, and immunity and reduce the toxicity of chemotherapy when compared with the chemotherapy alone.

All of the included studies reported “patients were randomised into two groups;” however, of 19 trials, only 3 studies described the randomization procedure in detail, and none of them mentioned blind and concealment of treatment allocation which is very important for rigorously designed RCTs. It has been shown that clinical studies using inadequate methods of ensuring allocation concealment are more likely associated with significant results than those using adequate concealment [41]. In addition, it is essential for the authors to describe how the participants who are lost to follow-up will be handled and whether those participants are monitored in survival analysis [42]. However, none of the included studies reported this information, and no trials mentioned whether they had used intention-to-treat (ITT) analysis. Thus, this meta-analysis indicated existence of potential risks of selection bias, performance bias, and detection bias, which would result in the overestimation of the clinical efficacy in the cinobufacini capsule plus chemotherapy group. Funnel plots and Egger's test indicated that there was potential risk of publication bias; however, we have demonstrated the conclusion was stable by searching conference abstracts, taking the heterogeneity test, and carrying out the trim and fill method. Altogether, the potential benefits of cinobufacini capsule for advanced NSCLC patients need to be further assessed through rigorously designed RCTs.

As a traditional Chinese medicine, cinobufacini, an aqueous extract from the parotid gland of Bufo toad, is widely used as an adjuvant therapy for various cancers [10, 43–46]. It has been reported that cinobufacini can reduce cell viability and induce genotoxicity and apoptosis in human lung cancer A549 cells in vitro [47, 48]. The extracted biologically active substance of cinobufacini mainly contains bufadienolides, alkaloids, and nucleosides [47, 49, 50]. It has been demonstrated that cinobufacini exerts the antitumor effect through many intracellular signaling mechanisms such as activating caspase-3 activity, inhibiting the expression of MAP kinase, and elevating Fas/Fasl and TNF-alpha/TNFR1 pathways [51, 52]. Resibufogenin, one of the components of cinobufacini, has been shown to inhibit the growth of A549 cells due to the degradation of cyclin D1 caused by the activation of glycogen synthase kinase-3*β* [53]. These biological characteristics of the active substance of cinobufacini might directly associate with the benefits to the NSCLC patients undergoing chemotherapy. Thus, these findings may provide evidence at the molecular level to support the clinical treatment with cinobufacini capsule for patients with NSCLC. However, to clarify the function of cinobufacin capsule as an adjunct to chemotherapy, future research focusing on the the bioactive components and specific mechanisms of cinobufacin capsule are needed. Furthermore, for future clinical research, it is essential to improve the methodological quality of RCTs and ensure that the reporting follows the CONSORT guidelines [42].

## 5. Conclusions

In summary, cinobufacini capsule combined with the first-line platinum-based chemotherapy regimen is superior to chemotherapy alone in the treatment of advanced NSCLC. However, the quality of the research included in this systematic review was not high, and the number of cases was small; this led to certain biases in the abovementioned conclusions. Therefore, it is necessary to design a large-scale, scientifically implemented large-scale study to strengthen the quality of research reports for the second evaluation. High-quality research studies should be provided to enhance the strength of the evidence in order to accurately guide clinical medication.

## Figures and Tables

**Table 1:** Principal characteristics of the studies included in the meta-analysis.

Study ID	Sample size(T/C)/case	Age (mean ± SD)		Stage	Intervention		Cinobufacini dose	Duration(day)	Outcome
		Test group	Control group		Test group	Control group			
Su [22]	44/44	56.48 ± 7.05	55.74 ± 6.12	IIIb, IV	DP + cinobufacini	DP	0.6 g·bid·po	84	①④⑤⑥
Li [23]	21/18	——	——	IIIb, IV	DP + cinobufacini	DP	0.75 g·tid·po	90	②
Chen et al. [24]	36/31	54.26 ± 10.38	53.45 ± 11.69	III, IV	GP + cinobufacini	GP	0.5 g·tid·po	84	①②③④⑤⑥⑦
Li [25]	38/37	60.09 ± 4.81	58.97 ± 4.63	III, IV	DP + cinobufacini	DP	0.5 g·tid·po	42	①⑥⑦
Huang [26]	43/43	61.58 ± 7.26	61.23 ± 7.15	IIIb, IV	GP + cinobufacini	GP	0.5 g·bid·po	42	①⑥⑦
Li et al. [23]	58/58	56.38 ± 8.24	57.12 ± 8.44	IIIb, IV	GP + cinobufacini	GP	0.5 g·bid·po	84	①③⑥⑦
Lan et al. [27]	43/42	55.63 ± 12.05	56.49 ± 11.61	III, IV	TC + cinobufacini	TC	0.5 g·tid·po	63	①
Chen et al. [28]	31/31	55.87 ± 6.58	56.29 ± 6.49	IIIb, IV	GP/AP + cinobufacini	GP/AP	0.5 g·tid·po	42	①⑤⑥⑦
Yu et al. [29]	33/30	——	——	III, IV	GP + cinobufacini	GP	0.5 g·tid·po	42	①④⑤⑥
Liu [30]	40/40	67.88 ± 2.27	68.54 ± 2.11	IIIb, IV	TP/NP + cinobufacini	TP/NP	0.6 g·bid·po	42	②④⑤⑥
Chen et al. [31]	36/36	56.90 ± 11.00	56.20 ± 10.70	IIIb, IV	TC + cinobufacini	TC	0.5 g·tid·po	84	①⑦
Pu et al. [32]	42/38	54.60 ± 9.38	57.83 ± 12.42	III, IV	GP + cinobufacini	GP	0.5 g·bid·po	42	①②③⑥⑦
Shi [33]	51/51	——	——	III, IV	TP + cinobufacini	TP	0.6 g·tid·po	42	①②④⑤⑥
Wei and Xu [34]	34/34	57.39 ± 4.31	58.31 ± 2.57	III, IV	GP + cinobufacini	GP	0.5 g·tid·po	42	①④⑥
Chen et al. [35]	40/40	59.30 ± 7.90	59.50 ± 7.50	IIIb, IV	GP + cinobufacini	GP	0.6 g·tid·po	42	①②③
Li et al. [36]	63/63	58.73 ± 9.54	57.96 ± 9.86	IIIb, IV	DP + cinobufacini	DP	0.5 g·tid·po	105	①②③④⑤⑥
Xiao et al. [37]	68/62	——	——	IV	GP + cinobufacini	GP	0.5 g·tid·po	42	①
Liu [38]	45/40	——	——	III, IV	NP/TP + cinobufacini	NP/TP	0.5 g·bid·po	42	①②③④⑤⑥
Miao et al. [39]	30/30	58.00 ± 7.00	57.00 ± 6.50	IIIb, IV	TP + cinobufacini	TP	0.5 g·tid·po	84	①④⑥

*Note*. (1) T is the test group; C is the control group; AP: pemetrexed + cisplatin; DP: docetaxel + cisplatin; GP: gemcitabine + cisplatin; NP: vinorelbine + cisplatin; TC: paclitaxel + carboplatin; TP: paclitaxel + cisplatin; (2) outcome index: ① ORR; ② one-year survival rate; ③ two-year survival rate; ④ leukocyte toxicity; ⑤ platelet toxicity; ⑥ vomiting reaction; ⑦ immune response.

**Table 2:** Grade system for grading the quality of evidence.

Quality assessment							No. of patients		Effect		Quality	Importance
No. of studies	Design	Risk of bias	Inconsistency	Indirectness	Imprecision	Other considerations	Meta-analysis	Control	Relative (95% CI)	Absolute		
*ORR*												
17	Randomised trials	Serious1	No serious inconsistency	No serious indirectness	No serious imprecision	None	416/735 (56.6%)	270/710 (38%)	RR 1.45 (1.3 to 1.61)	171 more per 1000 (from 114 more to 232 more)	⊕⊕⊕Omoderate	Critical
								36.7%		165 more per 1000 (from 110 more to 224 more)		

*One-year survive rate*												
8	Randomised trials	Serious1	No serious inconsistency	No serious indirectness	No serious imprecision	None	254/338 (75.1%)	178/321 (55.5%)	RR 1.35 (1.21 to 1.51)	194 more per 1000 (from 116 more to 283 more)	⊕⊕⊕Omoderate	Critical
								52.5%		184 more per 1000 (from 110 more to 268 more)		

*Two-year survive rate*												
6	Randomised trials	Serious1	No serious inconsistency	No serious indirectness	No serious imprecision	None	131/284 (46.1%)	70/270 (25.9%)	RR 1.78 (1.42 to 2.22)	202 more per 1000 (from 109 more to 316 more)	⊕⊕⊕Omoderate	Critical
								35%		273 more per 1000 (from 147 more to 427 more)		

*Leukocyte toxicity*												
9	Randomised trials	Serious1	No serious inconsistency	No serious indirectness	Serious2	None	167/376 (44.4%)	231/363 (63.6%)	RR 0.7 (0.62 to 0.79)	191 fewer per 1000 (from 134 fewer to 242 fewer)	⊕⊕OO low	Important
								66.7%		200 fewer per 1000 (from 140 fewer to 253 fewer)		

*Platelet toxicity*												
8	Randomised trials	Serious1	No serious inconsistency	No serious indirectness	Serious2	None	115/343 (33.5%)	166/333 (49.8%)	RR 0.67 (0.57 to 0.79)	165 fewer per 1000 (from 105 fewer to 214 fewer)	⊕⊕OO low	Important
								50.1%		165 fewer per 1000 (from 105 fewer to 215 fewer)		

*Vomiting toxicity*												
14	Randomised trials	No serious risk of bias	No serious inconsistency	No serious indirectness	No serious imprecision	Reporting bias3	260/588 (44.2%)	321/574 (55.9%)	RR 0.79 (0.7 to 0.88)	117 fewer per 1000 (from 67 fewer to 168 fewer)	⊕⊕⊕O moderate	Not important
								61.4%		129 fewer per 1000 (from 74 fewer to 184 fewer)		

*CD3* ^+^ *T cells (better indicated by lower values)*												
7	Randomised trials	No serious risk of bias1	No serious inconsistency	No serious indirectness	Serious2	Reporting bias3	284	274	—	SMD 1.42 higher (1.06 to 1.77 higher)	⊕⊕OO low	Important

*CD4* ^+^ *T cells (better indicated by lower values)*												
7	Randomised trials	No serious risk of bias	No serious inconsistency	No serious indirectness	Serious2	Reporting bias3	284	274	—	SMD 1.52 higher (1.22 to 1.83 higher)	⊕⊕OO low	Important

*CD4* ^+^ */CD8* ^+^ *T-cell ratio (better indicated by lower values)*												
7	Randomised trials	No serious risk of bias	No serious inconsistency	No serious indirectness	Serious2	Reporting bias3	284	274	—	SMD 1.34 higher (1.16 to 1.53 higher)	⊕⊕OO low	Important

^1^Risk of bias due to blind and random allocation concealment. ^2^Significant heterogeneity across studies. ^3^Small sample size.

**Table 3:** Principal characteristics of the studies included in the meta-analysis.

Outcome or subgroup		No. of Studies	Participants	Statistical method	Effect size	Heterogeneity	Publication bias (Egger's test)
ORR		17	1445	RR (fixed), 95% CI	1.49[1.33, 1.66]	*P*=0.994, *I*^2^ = 0.0%	*P*=0.001
One-year survival rate		8	659	RR (fixed), 95% CI	1.44[1.28, 1.63]	*P*=0.151, *I*^2^ = 34.7%	*P*=0.088
Two-year survival rate		6	554	RR (fixed), 95% CI	1.78[1.42, 2.22]	*P*=0.573, *I*^2^ = 0.0%	*P*=0.018
Leukocyte toxicity		9	739	RR (fixed), 95% CI	0.61[0.51, 0.72]	*P*=0.232, *I*^2^ = 24.7%	*P*=0.172
Platelet toxicity		8	676	RR (fixed), 95% CI	0.52[0.41, 0.67]	*P*=0.433, *I*^2^ = 0.0%	*P*=0.708
Vomiting response	Others + cinobufacini	8	678	RR (fixed), 95% CI	0.59[0.50, 0.69]	*P*=0.175, *I*^2^ = 31.7%	*P*=0.614
GP + cinobufacini		6	484	RR (fixed), 95% CI	1.12[0.96, 1.32]	*P*=0.466, *I*^2^ = 0.0%	*P*=0.811
CD3^+^level		7	558	SMD (fixed), 95% CI	1.25[1.05, 1.45]	*P*=0.874, *I*^2^ = 0.0%	*P*=0.883
CD4^+^level	84 days	3	255	SMD (fixed), 95% CI	1.88[1.58, 2.18]	*P*=0.314, *I*^2^ = 13.6%	*P*=0.765
	42 days	4	303	SMD (fixed), 95% CI	1.27[1.02, 1.51]	*P*=0.352, *I*^2^ = 8.1%	*P*=0.196
CD4^+^/CD8^+^level	0.5 g·tid·po	4	276	SMD (fixed), 95% CI	1.01[0.76, 1.26]	*P*=0.139, *I*^2^ = 45.4%	*P*=0.634
	0.5 g·bid·po	3	282	SMD (fixed), 95% CI	1.78[1.50, 2.06]	*P*=0.842, *I*^2^ = 0.0%	*P*=0.661

## Data Availability

The data used to support the finding of this study are available from the corresponding author upon request.
